# Promoter conservation in HDACs points to functional implications

**DOI:** 10.1186/s12864-019-5973-x

**Published:** 2019-07-27

**Authors:** Toni A. Boltz, Sawsan Khuri, Stefan Wuchty

**Affiliations:** 1 Department of Computer Science, University of Miami, Coral Gables, FL USA; 2 University of Exeter College of Medicine and Health, Exeter, UK; 3 Department of Biology, University of Miami, Coral Gables, FL USA; 4 Center of Computational Science, University of Miami, Coral Gables, FL USA; 5 Sylvester Comprehensive Cancer Center, University of Miami, Miami, FL USA; 6 Present address: University of California, Los Angeles, Los Angeles, CA USA

**Keywords:** HDAC, Promoter analysis, TFBS, Tissue specificity

## Abstract

**Background:**

Histone deacetylases (HDACs) are the proteins responsible for removing the acetyl group from lysine residues of core histones in chromosomes, a crucial component of gene regulation. Eleven known HDACs exist in humans and most other vertebrates. While the basic function of HDACs has been well characterized and new discoveries are still being made, the transcriptional regulation of their corresponding genes is still poorly understood.

**Results:**

Here, we conducted a computational analysis of the eleven HDAC promoter sequences in 25 vertebrate species to determine whether transcription factor binding sites (TFBSs) are conserved in HDAC evolution, and if so, whether they provide useful information about HDAC expression and function. Furthermore, we used tissue-specific information of transcription factors to investigate the potential expression patterns of HDACs in different human tissues based on their transcription factor binding sites. We found that the TFBS profiles of most of the HDACs were well conserved in closely related species for all HDAC promoters except HDAC7 and HDAC10. HDAC5 had particularly strong conservation across over half of the species studied, with nearly identical profiles in the primate species. Our comparisons of TFBSs with the tissue specific gene expression profiles of their corresponding TFs showed that most HDACs had the ability to be ubiquitously expressed. A few HDAC promoters exhibited the potential for preferential expression in certain tissues, most notably HDAC11 in gall bladder, while HDAC9 seemed to have less propensity for expression in the nervous system.

**Conclusions:**

In general, we found evolutionary conservation in HDAC promoters that seems to be more prominent for the ubiquitously expressed HDACs. In turn, when conservation did not follow usual phylogeny, human TFBS patterns indicated possible functional relevance. While we found that HDACs appear to uniformly expressed, we confirm that the functional differences in HDACs may be less a matter of location of activity than a question of which proteins and which acetyl groups they may be acting on.

**Electronic supplementary material:**

The online version of this article (10.1186/s12864-019-5973-x) contains supplementary material, which is available to authorized users.

## Background

Histone deacetylases (HDACs) remove the acetyl group from lysine residues of the N-terminal tail of core histones, allowing the repression of transcription. These metal binding proteins are mostly active in large multiprotein complexes, and can also act on non-histone proteins. Human histone deacetylases require zinc, and have been grouped into different classes based on their sequence similarity to homologues they have evolved from in yeast. Class I HDACs (1, 2, 3, 8) are most similar to yeast RPD3 protein, while Class II HDACs (4, 5, 6, 7, 9, 10) are homologues of yeast HDA1 [1]. HDAC11 forms Class IV on its own, sharing features from both Class I and Class II enzymes [1], while the sirtuin enzymes, which require NAD+ for catalysis and were formerly categorized as Class III HDACs, have evolved independently.

The HDAC proteins have been well characterized, and the position of their active site(s), their genomic position and cellular localization are well established (Table 1). Their modes of action have been investigated extensively over the last two decades, with particular emphasis on HDAC inhibitors as possible drugs for use in cancer therapy [4, 5]. However, the high level of similarity between the HDACs and the seemingly interchangeable nature of their activity makes them a complex family of proteins that has proven difficult to fully decipher [6].

**Table 1:** Genomic location, amino acid length of the main isoform, number of biochemically characterized active sites, and cellular localization of human histone deacetylases

		Chm	Protein length of main isoform (aa)	Number of active sites	Present in nucleus	Present in cytoplasm
Class I	HDAC1	1p34.1	482	1	P	N *(If present, results in axonal damage* [2]*)*
	HDAC2	6q21	488	1	P	N
	HDAC3	5q31.2	428	1	P	P
	HDAC8	Xq13	377	1	P	P [3]
Class IIA	HDAC4	2q37.2	1084	1	P	P
	HDAC5	17q21	1122	1	P	P
	HDAC7	12q13.1	952	1	P	P
	HDAC9	7p21.1	1011	1	P	P
Class IIB	HDAC6	Xp11.23	1215	2	P	P
	HDAC10	22q13.31	669	1	P	P
Class IV	HDAC11	3p25.1	347	1	P	P

Data from the NCBI Gene database, and localization data from [1], except where shown. *P* present, *N* Not present

Early studies on HDAC evolution found evidence of an ancient family of proteins with de-acetylase activity [7]. At the time, research focused on phylogenetic studies of protein sequences for the characterization of vertebrate HDAC active domains and localization signals to infer functional overlap and clues of alternative functions [8]. It was quickly ascertained that HDAC1 and HDAC2 are closely related and work in concert most of the time, and more recent work confirms that one is not a direct substitute for the other [9]. HDAC3 is equally widely expressed, interacts with Class II HDACs, and affects a wide range of cellular processes [10–12].

HDAC8, while also considered a Class I HDAC, seems to have evolved from a separate, equally ancient lineage that works on multiple substrates and is involved in several pathways [13, 14]. This histone deacetylase has a particular structure/function conformation that is different from its human homologues [15] and a propensity for de-fatty-acetylation [16]. Such findings suggest that some of the functional differences among HDACs may be linked to the nature of the acetyl compounds that they remove from their substrate proteins, which has profound implications on how we view and investigate this enzyme family.

Traditionally considered as recruiters for Class I HDACs due to their low catalytic activity when compared to other histone deacetylases [17], Class II HDACs 4, 5, 7, and 9 are now known to be active in their own right, playing a central role in regulating gene expression relating to muscle development, tissue differentiation and other pathways [18]. Extensive research has shown that HDAC4 is involved in a myriad of roles [19, 20], while HDAC5 is increasingly implicated in axon regeneration [21] and in cardiovascular contexts [6]. HDAC7 seems to play an important role for bone development [22] and in diabetes [23], while a flurry of recent articles have similarly associated HDAC9 with several disease pathways including various cancers and stroke [24–26].

HDACs 6 and 10 seem to have an interesting relationship and are often classified separately as Class IIB, as both have two highly similar catalytic domains, although the second domain of HDAC10 is considered inactive [1]. Comparative sequence analysis indicated that HDAC10 and HDAC6 may have shared a common ancestor at some point in vertebrate evolution [7], and ideas as to how both evolved separate functions are beginning to emerge. HDAC6 was the first histone deacetylase that was shown to work on a non-histone protein, tubulin, and is predominantly cytosolic [27], while recent work indicates that HDAC10 acts as a polyamine deacetylase [28]. Like other HDACs, both seem to be active in a variety of developmental and pathological contexts.

The only Class IV member, HDAC11, is arguably also the least well understood of the HDAC family. Recent reviews focus on its role in the immune system [29, 30], to the exclusion of other roles it may play that have not yet been discovered.

Given their vital and extensive roles in the regulation of gene expression and protein activity in eukaryotic genomes both in and out of the nucleus, relatively little is known about the regulation of HDAC expression. We expect that the transcription of HDACs does not differ markedly from other genes whose promoters are regulated by histone phosphorylation and acetylation [31], which they, as *the* histone deacetylases, are necessarily involved in [32]. Regulation of HDACs in cancer cells by the ubiquitous transcription factors Sp1 and Sp3 has been well investigated [33], and their expression profile has been studied in some disease cases [34, 35]. Furthermore, HDACs are subjected to the same array of post-translational modification as other proteins [36]. Increasing evidence is being accumulated about HDAC roles in development, housekeeping functions, and disease onset and progression, none the least in cancers. In plants, histone deacetylases have been shown to act on a wide array of molecules, including *N*-acetyleserotonin [37].

Despite our expanding knowledge, there seems to be an urgent need to elucidate their separate functions and intersections of function, and to better understand how their own expression is regulated. Turning to computational methods as potential guides to bench experimentation, we conducted an in-depth analysis of HDAC promoter sequences with two questions in mind: Are transcription factor binding sites (TFBSs) conserved in HDAC evolution, and if so, do they provide useful information about HDAC transcriptional regulation and HDAC function?

These questions were fueled by recent literature on the slow evolution of TFBSs [38, 39] and their potential use in highlighting gene expression patterns (reviewed in [40]). Given that there is no gold standard to assess methods for TF analysis [41], and as divergent as promoter sequences can be among closely related species (e.g. [42]) and among the promoter regions of closely related genes (e.g. [43]), there seems to be enough signal in them to imply functional relevance [39] which can then be confirmed by experimental data. Given the functional overlap that HDACs seem to have, and increasing evidence of their ubiquity in the human system, we were curious whether there were any signals in their promoters which could help deepen our understanding of this enzyme family.

## Results

### Evolutionary conservation of TFBSs in HDAC promoters

**Fig. 1:**
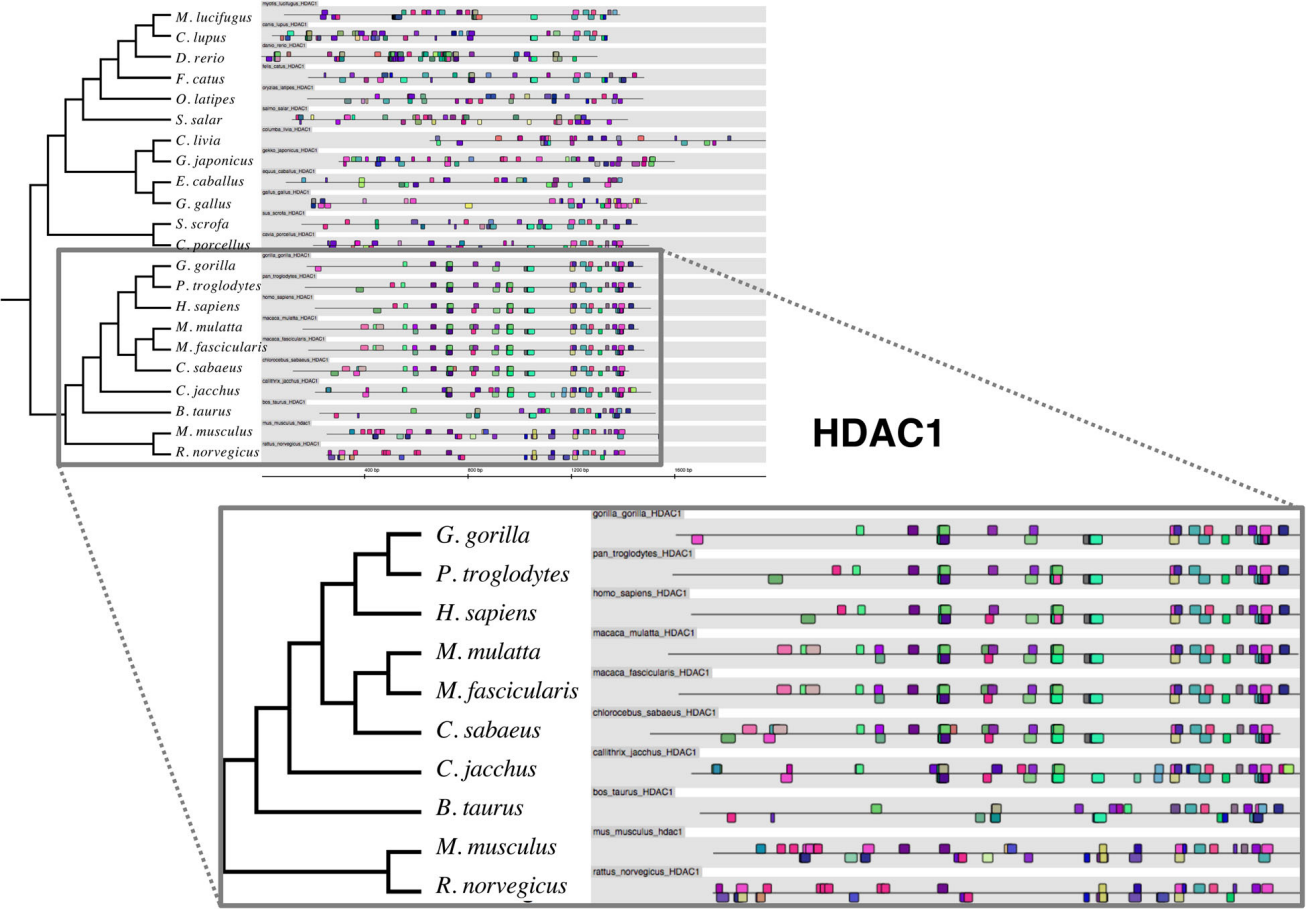
Evolutionary conservation of transcription factor binding sites in HDAC1 promoter sequences from different organisms. The TFBS pattern of promoter sequences of HDAC1 in 22 different organisms, results as shown in Genomatix. As expected, TFBS patterns in *H. sapiens* are strongly conserved in closely related primate organisms, including *G. gorilla*, *P. troglodytes*, *M. mulatta* and *M. fascicularis* (box)

We found that human TFBS patterns in HDAC promoters are evolutionarily conserved across all HDACs, with only HDACs 5, 7 and 10 indicating unusual patterns of TFBS distribution along the promoter region. In Fig. 1 we present the HDAC1 promoter alignment as an example of the Genomatix output, showing promoter sequences aligned according to the quantitative phylogenetic distances between their TFBS patterns. Here, the TFBS patterns appeared in the predictable evolutionary groupings, with apes (*H. sapiens*, *G. gorilla* and *P. troglodytes*) and rodents (*R. norvegicus* and *M. musculus*) forming clades. We observed similar patterns in HDACs 2, 3, 4, 6, 8, 9 and in HDAC11 (Additional file 2: Figure S1, Additional file 3: Figure S2, Additional file 4: Figure S3, Additional file 5: Figure S4, Additional file 6: Figure S5, Additional file 7: Figure S6 and Additional file 8: Figure S7). HDACs 8 and 9 had evolutionarily conserved TFBS patterns in closely related organisms (Additional file 6: Figure S5, Additional file 7: Figure S6). Notably, a HDAC8 equivalent was absent in *P. troglodytes* while an HDAC9 equivalent was missing from *G. gorilla*.

**Fig. 2:**
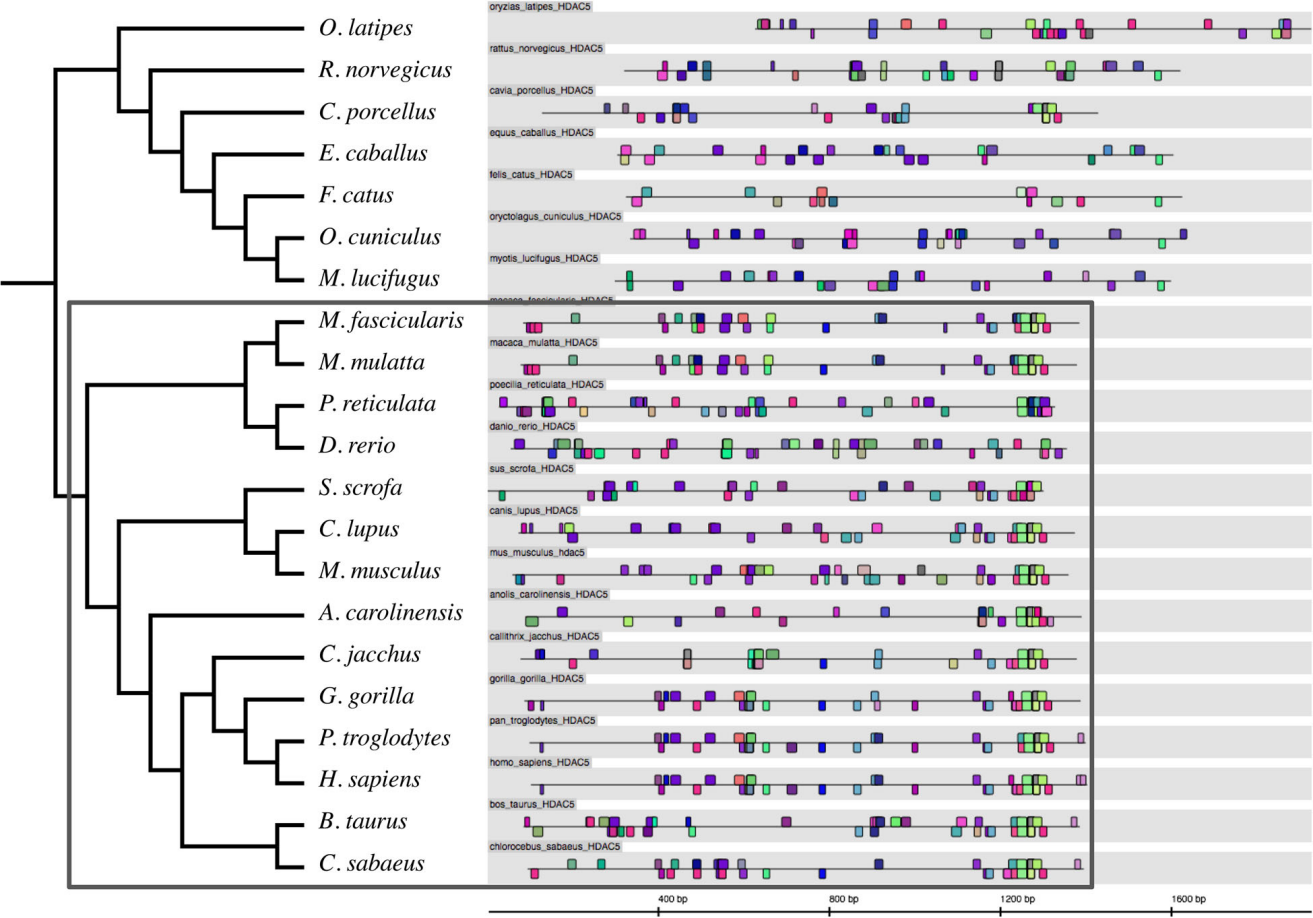
Patterns of transcription factor binding sites in HDAC5 promoter sequences in different organisms. The TFBS pattern of promoter sequences of HDAC5 in 21 different organisms, results as shown in Genomatix. The TFBS pattern of promoter sequences of HDAC5 shows high similarity across species, of note is the subtree that includes *M. fascicularis* and *C. sabaeus*, especially near the transcriptional start site. As expected, *H. sapiens* occurs within the same clade as *P. troglodytes* and *G. gorilla*

**Fig. 3:**
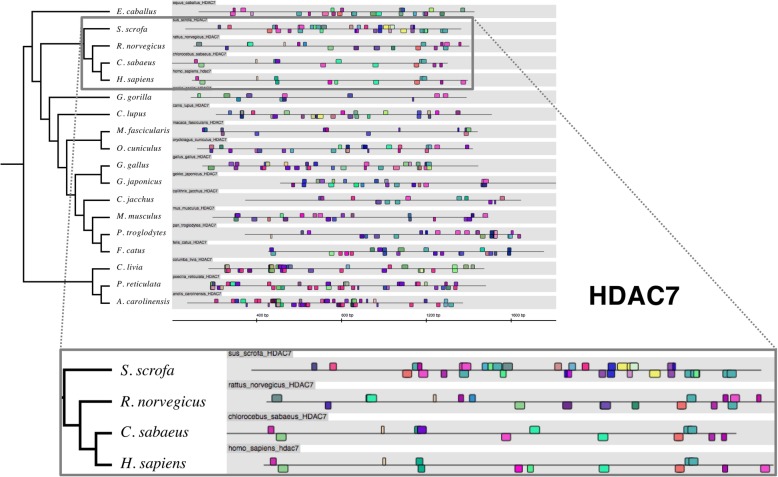
Patterns of transcription factor binding sites in HDAC7 promoter sequences in different organisms. The TFBS pattern of promoter sequences of HDAC7 in 18 different organisms, results as shown in Genomatix. Although TFBS patterns of the *H. sapiens* sequence shows similarity with that of green monkey (*C. sabaeus*, box), little overall conservation of TFBS patterns in the HDAC7 promoters exists

**Fig. 4:**
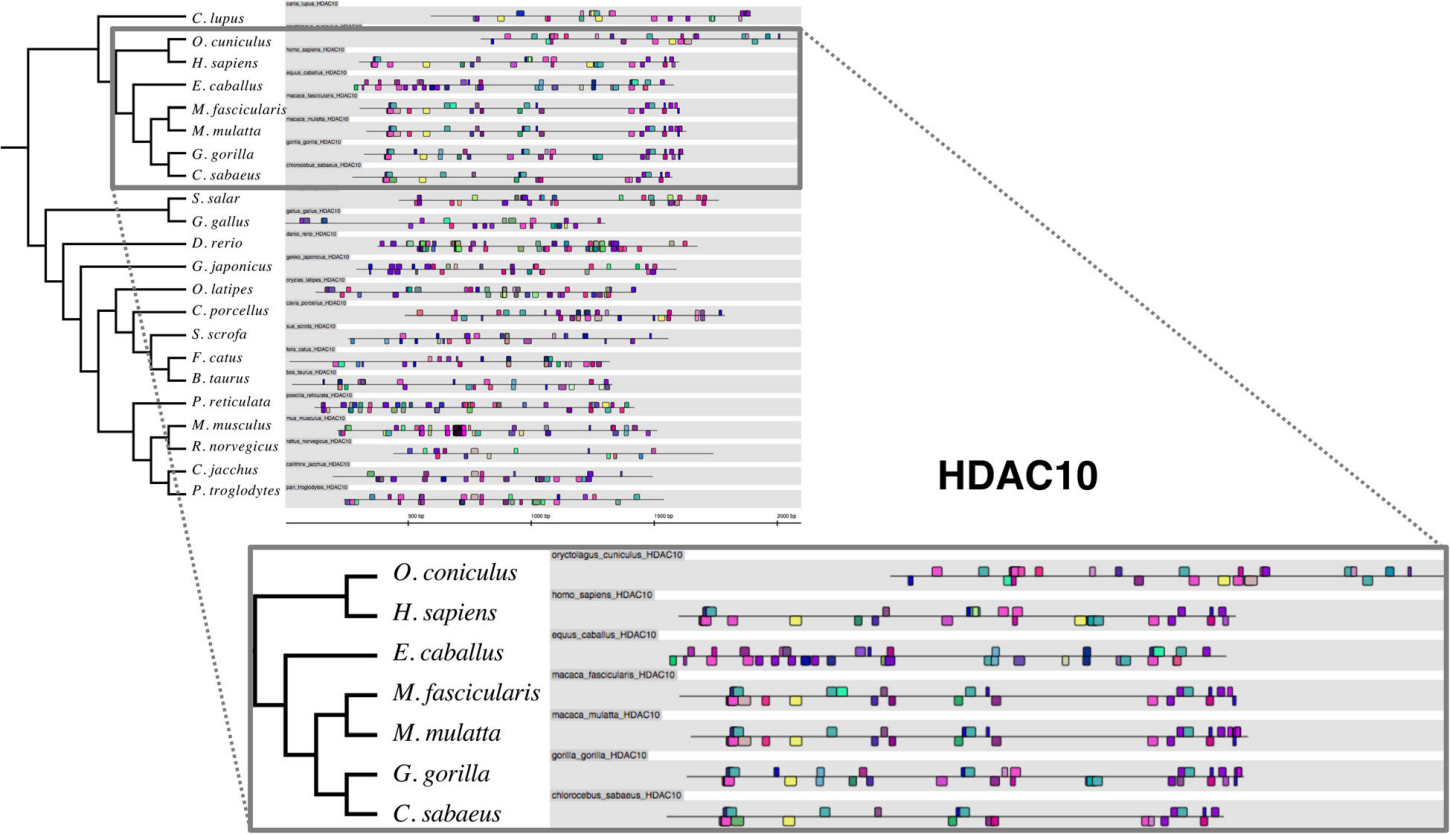
Evolutionary conservation of transcription factor binding sites in HDAC10 promoter sequences in different organisms. The TFBS pattern of promoter sequences of HDAC10 in 22 different organisms, results as shown in Genomatix. We found an unexpected pattern of conservation where *O. cuniculus* and *E. caballus* promoter patterns were unusually similar to those of primates (box)

The promoter of HDAC5 was conserved for the greatest number of species (Fig. 2), and had two fish species (*P. reticulata* and *D. rerio*) clustering close to two old world monkeys, while promoters of HDAC5 homologues from rat and mouse occupied different ends of the dendrogram. Promoter sequences of HDACs 7 and 10 also showed transcription factor binding site patterns where the classic phylogenetic lineages did not hold true. The TFBS profile of human HDAC7 (Fig. 3) appeared to be most similar to that of pig *S. scrofa*, green monkey *C. sabaeus* and rat *R. norvegicus*, while the TFBSs from other primate promoters appeared to follow different patterns. The promoter of human HDAC10 (Fig. 4) was most similar to that of rabbit, *O. cuniculus*, while the promoter sequences of other primate HDAC10s were more similar to that from horse, *E. caballus*.

### TFBS patterns provide useful information on HDAC regulation and function

We analyzed the large amount of gene expression data available from mRNA studies [44] and found that most HDACs seemed to be able to be expressed in most tissues, albeit showing higher expression levels in some tissues than in others. For the most part, these expression profiles were based on experiments that were not targeting HDAC function per se, suggesting that drawing patterns of tissue specificity from these results may be challenging.

Using the previously gathered promoter motif data in a new context, we considered transcription factor binding site trimers that were present on HDAC promoters to denote expression in a given tissue. In particular, we used a non-exclusive set defined as TFs expressed in most tissues, and a preferentially expressed set of TFs that were more highly expressed in the given tissues when compared to other tissues found in Genomatix (as described in Methods). In particular, a trimer was only considered if all 3 units - a unit being the transcription factor whose binding site is present on the relevant HDAC promoter - were expressed within the given tissue, pointing to evidence that the corresponding HDAC is expressed in the underlying tissue. To help identify overarching patterns, we collapsed the 59 non-disease tissue specificity designations available into the 11 human biological systems (Additional file 1: Table S1), namely cardiovascular/hematopoietic, digestive, endocrine, excretory, immune/lymphatic, integumentary, muscular, nervous, respiratory, reproductive, and skeletal systems, with an additional designation for embryonic expression. In Table 2, we list the biological systems where we found at least one TFBS trimer that appeared in a human HDAC promoter sequence, and compared these results to previously reported tissue specificity of HDACs. Since we only used human data in this analysis, the absence of a TFBS trimer on an HDAC promoter could indicate that this HDAC is not expressed in the corresponding tissue, indicated in Table 2 using an “All Except” annotation.

**Table 2:** Tissue and system specificity of histone deacetylases. Reported data is from cited references [5, 17, 45–47]. Predicted data is based on enrichment of TFBS trimers in the analysis described in the main text. Following TF groupings in Genomatix, *non-exclusive* refers to the presence of TFs that are active in most tissues, and *preferential* refers to TFs that are more highly expressed in these systems than in others. Dark bullet points denote predicted activity, outline bullet points denote a predicted lack of activity in these systems

		Reported tissue specificity	Predicted non-exclusive system specificity	Predicted preferential system specificity
Class I	HDAC1	Ubiquitous	All	All
	HDAC2	Ubiquitous	All Except o Respiratory	• Cardiovascular/Hematopoietic • Embryo • Endocrine • Immune/Lymphatic • Muscular • Nervous
	HDAC3	Ubiquitous	All Except o Integumentary o Respiratory	• Cardiovascular/Hematopoietic • Immune/Lymphatic • Muscular
	HDAC8	• Muscular (smooth muscle)	All	All
Class IIA	HDAC4	• Cardiovascular/Hematopoietic • Muscular (smooth muscle) • Nervous (brain) • Endocrine (liver)	All	All Except o Respiratory
	HDAC5	• Cardiovascular/Hematopoietic • Muscular (smooth muscle) • Nervous (brain) • Endocrine (liver)	All Except o Respiratory	All Except o Integumentary o Respiratory o Skeletal
	HDAC7	• Cardiovascular/Hematopoietic • Muscular (smooth muscle) • Endocrine (several tissues) • Skeletal	• Cardiovascular/Hematopoietic • Digestive • Endocrine • Excretory • Immune/Lymphatic • Muscle • Nervous	• Cardiovascular/Hematopoietic • Digestive • Endocrine • Excretory • Muscle • Nervous
	HDAC9	• Immune/Lymphatic • Muscular (smooth muscle) • Nervous (brain)	All Except o Digestive o Excretory o Integumentary o Respiratory	• Cardiovascular/Hematopoietic • Embryo • Immune/Lymphatic • Muscular
Class IIB	HDAC6	• Cardiovascular/Hematopoietic • Endocrine (liver, pancreas) • Nervous (brain)	All Except o Respiratory	All Except o Embryo o Respiratory
	HDAC10	• Endocrine (kidney, liver, spleen) • Excretory (kidney)	All Except o Integumentary o Respiratory o Skeletal	• Cardiovascular/Hematopoietic • Endocrine • Immune/Lymphatic • Muscular
Class IV	HDAC11	• Cardiovascular/Hematopoietic • Endocrine (kidney) • Excretory (kidney) • Muscular (smooth muscle) • Nervous (brain)	• Cardiovascular/Hematopoietic • Digestive • Endocrine • Excretory • Embryo • Immune/Lymphatic • Nervous • Reproductive	Not enough TF trimers for a signal

Class I HDACs 1, 2, and 3 are particularly well-studied and known to be ubiquitously expressed [9–12], validating our approach. HDAC8, known to be active on multiple substrates [15] and many different proteins [48] is only reported to be highly expressed in smooth muscle, while our results suggest that HDAC8 is another ubiquitous HDAC (Table 2). In fact, when non-exclusive TFs were included in this trimer analysis, almost all of the HDACs had fairly widespread tissue representation, with the exception of HDACs 7 and 11, which had fewer tissues represented in the results, suggesting a narrower expression range. When only preferentially expressed TFs were considered, HDAC3 had TFBS trimers from only 3 major systems such as cardiovascular/hematopoietic, immune/lymphatic, and muscular. HDACs 9 and 10 also had these same three major systems represented as TFBS trimers in their promoters, in addition to the embryonic system in HDAC9 and the endocrine system in HDAC10 promoters. The promoter sequence of HDAC11 had no trimers present when the preferential expression filter was applied, suggesting that its expression is governed only through non-exclusive TFs.

**Fig. 5:**
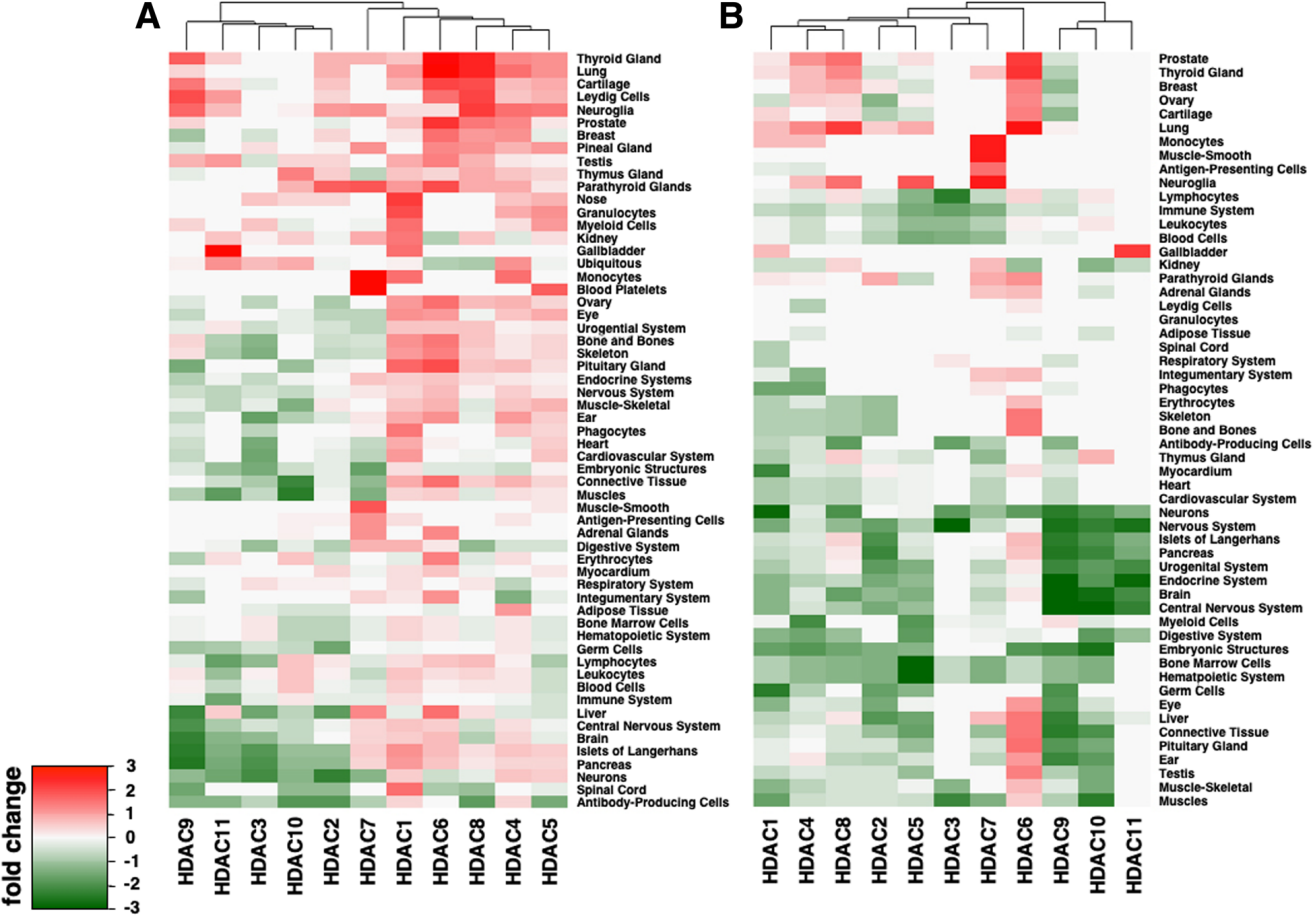
Enrichment of tissue type TFBSs in each HDAC promoter. Showing the log-fold change of observed and expected TFBS frequencies in 59 different tissues we assessed the prevalence of TFBSs on HDAC promoters that correspond to TFs that are expressed in a tissue specific manner. In particular, we consider the enrichment/dilution of TFBSs through a (**a**) non-exclusive set defined as TFs which were expressed in most tissues, and (**b**) a preferentially expressed set of TFs that were more highly expressed in the given tissues when compared to other tissues

This analysis of trimers also indicated that there were higher instances of TFs across the HDACs that were expressed in the nervous, immune and endocrine systems. However, this observation may reflect the fact that there were disproportionately more TFs listed under these systems in the Genomatix annotation than for other systems, such as respiratory or integumentary. Therefore, we fine-tuned our approach, treating TFBSs individually and determining the observed numbers of binding sites of TFs that appear in HDAC promoter sequence and are expressed in specific tissues, and used a log_2_-fold change to assess the significance of our findings (see Methods) when we compared observed to expected numbers. In the heatmap in Fig. 5a where we considered a non-exclusive set of TFs that were expressed in most tissues, we observed that HDACs 1,4,5,6 and 8 appear to be largely associated to the majority of 59 tissues. In turn, HDACs 2,3,9,10 and 11 seem to be less likely expressed in most tissues. In particular, HDAC7 seems to be associated with expression in blood cells, and less in the embryonic system, while TFBSs for TFs associated with the nervous system seem to be underrepresented in the promoter of HDAC9. Furthermore, HDACs 6 and 8 appear strongly over-represented in thyroid gland, lung and cartilage. HDAC10 had a low score for TFBSs associated with muscle tissue, while HDAC11 had a high score for TFBSs associated with the gall bladder.

As for the heatmap in Fig. 5b, where we considered preferentially expressed set of TFs that were more highly expressed in given than other tissues we observed a high degree of paucity of binding sites on HDAC promoters. Specifically, we found enrichment for TFBSs associated with the nervous system in the HDAC3 promoter, and for those associated specifically with neuralgia and smooth muscle in the promoter of HDAC7. Similar to the trimer results, we observed fewer TFs associated with the nervous system on the promoter of HDAC9, while the high specificity for expression in gall bladder remained highlighted in the promoter region for HDAC11.

## Discussion

In this work, we were curious whether there was any evolutionary conservation in HDAC promoter sequences, and if so what it can tell us about the transcriptional regulation and function of the eleven human HDACs. Our results confirmed that in general, there was evolutionary conservation in HDAC promoters, and in cases where this conservation did not align with currently accepted phylogeny, the pattern of TFBS arrangement on human promoters showed some similarity with different species, indicating a possible functional relevance. Unusual patterns in genetic sequence phylogenies suggest dynamic and relatively recent changes in the evolution of such sequences, implying evolutionarily recent patterns in the way the corresponding HDACs are regulated [49]. In fact, promoter conservation among vertebrate species seems to be more prominent for the ubiquitously expressed HDACs, particularly for HDACs 1 and 2, suggesting that these have not undergone recent evolution, a hypothesis in line with literature on the evolution of so-called “housekeeping” and “essential” genes [50]. Those HDACs that exhibit an unusual pattern of TFBSs on their promoters seem to also have a propensity for expression in fewer tissues such as seen in our results of HDAC5 and their possible preferential association with the cardiovascular/hematopoietic, muscular, nervous and endocrine tissues (Table 2). The exceptional case in our results is HDAC11 that has a conserved promoter region which followed conventional species phylogenies. Yet, HDAC11 also exhibited a possible preferential association with expression in the gall bladder, a conclusion we recommend to be followed up with laboratory experimentation.

Overall, our results imply that most HDACs are able to be ubiquitously expressed. For example, our results of the HDAC8 promoter region concur with studies into the evolution of HDAC catalytic domains, highlighting the relatively recent functional evolution of HDAC8 [8]. In turn, these are validated by recent discoveries about HDAC8 function [15, 16, 51] which further indicate that the differences between the HDACs may not be one of location of expression as much as a structure/function difference in their catalytic process.

Early phylogenetic studies using HDAC protein sequences did not report differences between the catalytic domain of HDAC7 and the other Class II HDACs [8]. Our findings showed that there was little evolutionary conservation in the promoter sequence of HDAC7, and that it has a broad tissue specificity spanning most biological systems but perhaps not as strongly in embryonic tissues. Recent molecular investigations place HDAC7 in the endocrine and skeletal systems [23] as well as in the brain playing a key role in memory formation [52]. Like HDAC8, the regulation of HDAC7 seems to have evolved differently to its functional relevance, making it particularly interesting for further experimental investigations similar to those that have taken place in HDAC8 as detailed above.

## Conclusions

Previous studies have shown that quantitative differences in transcription factor binding are observable even in closely related species, yet only a weak correlation is found between binding variation and regulatory function [53]. Furthermore, TFBSs have a high evolutionary turnover rate such that even closely related species may not have conserved binding sites on their promoters [54]. This may explain some of the differences we observed in TFBS pattern along the promoters of HDACs from different species. Given that low evolutionary conservation at the promoter level may not have a significant effect on gene expression [54, 55], we posit that our two-pronged approach points to new avenues for studying the regulation of HDAC expression. Since the HDACs themselves are heavily involved in gene expression, further studies into the transcriptomic levels of these genes may prove useful to compare to TFBS patterns reported here. This will allow us to infer how sensitive the regulation of regulatory proteins is with regards to differences at both the binding site and transcriptomic level.

Exploring the biochemical role of each of the HDAC homologues in the different species we tested would shed further light as to how these proteins have evolved, and why. Early thoughts about why there are eleven HDACs in the human system had focused on tissue or time specificity, and there is enough information about their function now to add several layers of complexity on this question*.* Our results suggest that all HDACs are ubiquitously expressed, and that the differences between them rest in which acetyl group they remove from a protein, and which proteins they act on, instead of where they act. Given their role in gene expression and the impact that dysregulation of HDACs can have on the health of an organism, it is crucial that a comprehensive analysis of the biochemical roles and transcriptional regulation of these enzymes is performed, so that better targeted therapeutics can be identified. With HDAC inhibitors gaining traction in the treatment of various cancers and other diseases, the ability to fully understand their regulation and function, including through experimental promoter validation, remains a crucial research priority.

## Methods

### Transcription factor binding sites

To determine TFBSs in the promoters of human HDAC genes, we considered promoter sequences starting from 1,200 base pairs upstream to 100 base pairs downstream of the transcriptional start site (TSS) as designated in the National Center for Biotechnology Information’s (NCBI) Nucleotide database. Research in yeast found that conservation of TFBSs is highest within 200 bp upstream of the TSS [42]. Furthermore, there is evidence of multiple TSSs and alternative promoters per HDAC according to the Database for Transcriptional Start Sites (DBTSS) [56]. We therefore considered a range of 1,200 bp upstream of the NCBI’s TSS to capture as many TFBS signals as might be present from possible alternative TSSs.

We extracted known and annotated HDAC sequences in 25 different species from the NCBI’s Nucleotide database. To check their similarity and possible kinship we aligned organism-specific HDACs with their human counterparts. In particular, we established 11 HDAC groups of sequences that are annotated according to their corresponding human HDAC. Although we only considered known organism-specific HDACs, we reviewed their annotations and assigned a given organism-specific HDAC to the corresponding group, if the corresponding sequence was most similar to the underlying human HDAC. We then extracted their promoter sequences for our analysis using the Genomatix software suite (www.genomatix.com), as its transcription factor database has a taxonomically relevant classification system that was applicable to all the considered species. The computational detection of TFBS motifs was based on scanning these promoter sequences through position weight matrices of corresponding transcription factors with MatInspector as implemented in the Genomatix software suite [57, 58], that was also used to visualize TFBSs on the promoter sequences. We set a core similarity of 0.75 (maximum is 1.0) and a matrix similarity of the optimized value + 0.10 to find TFBSs. We used transcription factor motifs from transcription factor families that were found in either all species or only in vertebrates.

### Similarity of TFBS profiles

Every promoter is initially represented by a sequence of TFBSs. We normalized the presence of 3-mers (trimers) of TFBSs by $p\left({\alpha}_1,{\alpha}_2,{\alpha}_3\ \right)=\frac{f\left({\alpha}_1,{\alpha}_2,{\alpha}_3\right)}{L-2}$ where *L* is the number of binding sites on the promoter, and *α*_*i*_ refers to a particular transcription factor. Randomness in this data was reduced via the corresponding 2-mers and 1-mers through

$$ {p}^0\left({\alpha}_1,{\alpha}_2,{\alpha}_3\ \right)=\frac{p\left({\alpha}_1,{\alpha}_2\right)p\left({\alpha}_2,{\alpha}_3\right)}{p\left({\alpha}_2\right)}. $$

In a promoter sequence we determined the occurrence of a 3-mer *m* of TFBSs as

$$ m\left({\alpha}_1,{\alpha}_2,{\alpha}_3\ \right)=\left\{\begin{array}{c}\frac{p\left({\alpha}_1,{\alpha}_2,{\alpha}_3\right)-{p}^0\left({\alpha}_1,{\alpha}_2,{\alpha}_3\ \right)}{p^0\left({\alpha}_1,{\alpha}_2,{\alpha}_3\ \right)}\\ {}0\end{array}\right.\mathrm{if}\ {\displaystyle \begin{array}{c}{p}^0\ne 0\\ {}{p}^0=0\end{array}}. $$

As a consequence, each promoter sequence was represented as a profile of trimers.

Comparing pairs of trimer profiles of TFBSs between species, we defined a distance between promoter sequences *M* and *N* as the cosine distance between profiles of transcription factor binding sites as

$$ D\left(M,N\right)=1-\frac{\sum_{i=1}^k{m}_i\times {n}_i}{\sqrt{\sum_{i=1}^k{m}_i^2}\sqrt{\sum_{i=1}^k{n}_i^2}}. $$

This similarity measure was used to determine all pairwise distances between promoter profiles of TFBSs between species. Distance matrices were used to reconstruct the trees using the neighbor-joining algorithm as implemented in the DendroPy Phylogenetic Computing Library [59]. The resulting dendrograms were visualized using FigTree, a freely available web-based software tool (http://tree.bio.ed.ac.uk/software/figtree/).

### Tissue specific TFBSs

The Genomatix database [57, 58] was again used to determine the names and descriptions of the transcription factors as well as their recorded tissue-specific expression. According to Genomatix, “the tissue associations of matrix families are determined by automatic evaluation of all PubMed abstracts (co-citations of transcription factors and tissues) and subsequent manual curation.” Specifically, we considered a non-exclusive set defined as TFs expressed in most tissues, and a preferentially expressed set of TFs that were more highly expressed in the given tissues when compared to other tissues. Only human promoters were used for tissue specificity analysis, due to availability of data.

As a general expected value for transcription factors that appear on a given promoter *p* and are expressed in a tissue *t*, *E*_*p,t*_, we defined ${E}_{p,t}=\frac{x_t}{\left|\bigcup \limits_t{x}_t\right|}{n}_p$, where *x*_*t*_ is the number of transcription factors that are expressed in tissue *t*, while $\left|\bigcup \limits_t{x}_t\right|$ is the total number of transcription factors in all tissues, and *n*_*p*_ is the number of transcription factor binding sites in the underlying promoters sequence *p*. We utilized this background distribution to determine the enrichment of a promoter sequence *p* in a tissue *t*, defined as ${f}_{p,t}=\frac{O_{p,t}}{E_{p,t}}$, where *O*_*p,t*_ is the observed number of transcription factor binding sites that appear in promoter sequence *p* and are expressed in tissue *t*. Given the difficulty of assessing significance in this domain, and the lack of direct activity data of TFs on HDAC promoters [41], we considered the log_2_-fold change of observed and expected TFBS frequencies ${fc}_{p,t}={\log}_2\frac{O_{p,t}}{E_{p,t}}$ which allowed us to assess the prevalence of expressed TFBSs that occur in a given HDAC promoter sequence in a given tissue. As a consequence, a promoter sequence appears enriched in a given tissue if *fc*_*pt*_ > 1 and diluted if *fc*_*pt*_ < − 1.

## Additional files

Additional file 1 : **Table S1** Tissue to System classification. (DOCX 17 kb)

Additional file 2 : **Figure S1** Evolutionary conservation of transcription factor binding sites in HDAC2 promoter sequences in different organisms. (DOCX 3802 kb)

Additional file 3 : **Figure S2** Evolutionary conservation of transcription factor binding sites in HDAC3 promoter sequences in different organisms. (DOCX 3534 kb)

Additional file 4 : **Figure S3** Evolutionary conservation of transcription factor binding sites in HDAC4 promoter sequences in different organisms. (DOCX 3746 kb)

Additional file 5 : **Figure S4** Evolutionary conservation of transcription factor binding sites in HDAC6 promoter sequences in different organisms. (DOCX 4280 kb)

Additional file 6 : **Figure S5** Evolutionary conservation of transcription factor binding sites in HDAC8 promoter sequences in different organisms. (DOCX 3673 kb)

Additional file 7 : **Figure S6** Evolutionary conservation of transcription factor binding sites in HDAC9 promoter sequences in different organisms. (DOCX 3052 kb)

Additional file 8 : **Figure S7** Evolutionary conservation of transcription factor binding sites in HDAC11 promoter sequences in different organisms. (DOCX 3544 kb)

## Data Availability

All used data was obtained from the databases referenced. Tissue specific and transcription factor binding site data was obtained from Genomatix (www.genomatix.com).

## Abbreviations

HDAC: Histone deacetylases
TFBS: Transcription factor binding site
