# Supplementary material for: Promoter conservation in HDACs points to functional implications
Source: BMC Genomics. 2019 Jul 27;20:613. doi: 10.1186/s12864-019-5973-x (PMC6660948; doi:10.1186/s12864-019-5973-x)
Supplement: Supplementary file 1 — : Table S1 Tissue to System classification. (DOCX 17 kb) [file 12864_2019_5973_MOESM1_ESM.docx]

**Table S1.** Tissue to System classification.

| Tissues | System |
| --- | --- |
| Blood Cells | Cardiovascular/Hematopoietic |
| Blood Platelets | Cardiovascular/Hematopoietic |
| Cardiovascular System | Cardiovascular/Hematopoietic |
| Erythrocytes | Cardiovascular/Hematopoietic |
| Granulocytes | Cardiovascular/Hematopoietic |
| Heart | Cardiovascular/Hematopoietic |
| Hematopoietic System | Cardiovascular/Hematopoietic |
| Hemocytes | Cardiovascular/Hematopoietic |
| Leukocytes | Cardiovascular/Hematopoietic |
| Myocardium | Cardiovascular/Hematopoietic |
| Digestive System | Digestive/Excretory |
| Gallbladder | Digestive/Excretory |
| Liver | Digestive/Excretory |
| Embryonic Structures | Embryo |
| Adrenal Glands | Endocrine |
| Endocrine System | Endocrine |
| Islets of Langerhans | Endocrine |
| Pancreas | Endocrine |
| Parathyroid Glands | Endocrine |
| Pituitary Gland | Endocrine |
| Thyroid Gland | Endocrine |
| Antibody-Producing Cells | Immune/Lymphatic |
| Antigen-Presenting Cells | Immune/Lymphatic |
| Bone Marrow Cells | Immune/Lymphatic |
| Germ Cells | Immune/Lymphatic |
| Immune System | Immune/Lymphatic |
| Lymphocytes | Immune/Lymphatic |
| Monocytes | Immune/Lymphatic |
| Phagocytes | Immune/Lymphatic |
| Thymus Gland | Immune/Lymphatic |
| Adipose Tissue | Integumentary |
| Breast | Integumentary |
| Integumentary System | Integumentary |
| Muscle, Skeletal | Muscular |
| Muscle, Smooth | Muscular |
| Muscles | Muscular |
| Myeloid Cells | Muscular |
| Brain | Nervous |
| Central Nervous System | Nervous |
| Ear | Nervous |
| Eye | Nervous |
| Nervous System | Nervous |
| Neuroglia | Nervous |
| Neurons | Nervous |
| Nose | Nervous |
| Pineal Gland | Nervous |
| Spinal Cord | Nervous |
| Lung | Respiratory |
| Respiratory System | Respiratory |
| Bone and Bones | Skeletal |
| Cartilage | Skeletal |
| Connective Tissue | Skeletal |
| Skeleton | Skeletal |
| Ubiquitous | Ubiquitous |
| Bladder | Uro-Reproductive |
| Blastomeres | Uro-Reproductive |
| Kidney | Uro-Reproductive |
| Leydig Cells | Uro-Reproductive |
| Luteal Cells | Uro-Reproductive |
| Ovary | Uro-Reproductive |
| Prostate | Uro-Reproductive |
| Testis | Uro-Reproductive |
| Urogenital System | Uro-Reproductive |
