# Supplementary material for: Promoter conservation in HDACs points to functional implications
Source: BMC Genomics. 2019 Jul 27;20:613. doi: 10.1186/s12864-019-5973-x (PMC6660948; doi:10.1186/s12864-019-5973-x)
Supplement: Supplementary file 2 — : Figure S1 Evolutionary conservation of transcription factor binding sites in HDAC2 promoter sequences in different organisms. (DOCX 3802 kb) [file 12864_2019_5973_MOESM2_ESM.docx]

**
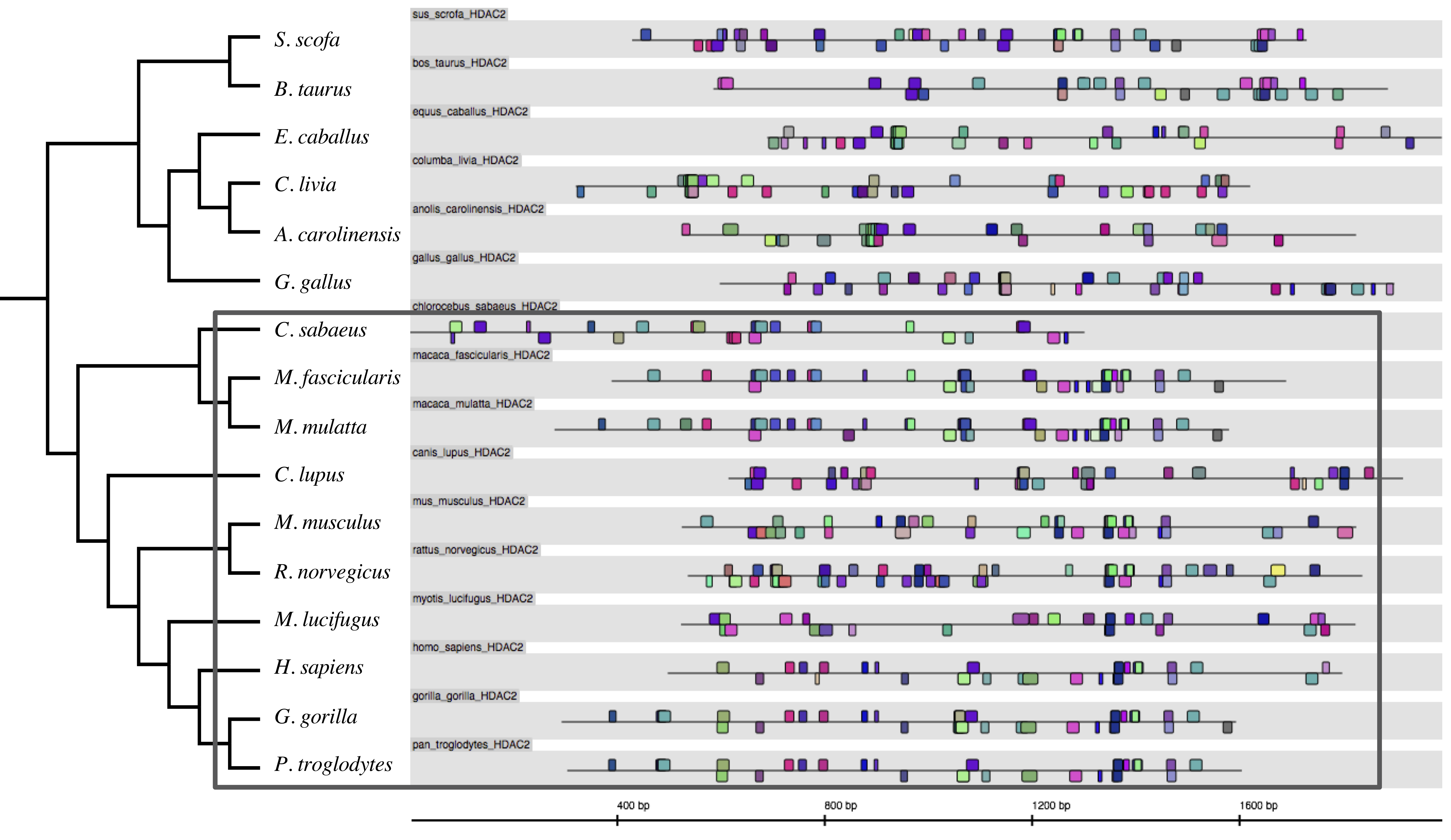
Figure S1.** Evolutionary conservation of transcription factor binding sites in HDAC2 promoter sequences in different organisms.
