# Supplementary material for: Promoter conservation in HDACs points to functional implications
Source: BMC Genomics. 2019 Jul 27;20:613. doi: 10.1186/s12864-019-5973-x (PMC6660948; doi:10.1186/s12864-019-5973-x)
Supplement: Supplementary file 4 — : Figure S3 Evolutionary conservation of transcription factor binding sites in HDAC4 promoter sequences in different organisms. (DOCX 3746 kb) [file 12864_2019_5973_MOESM4_ESM.docx]

**
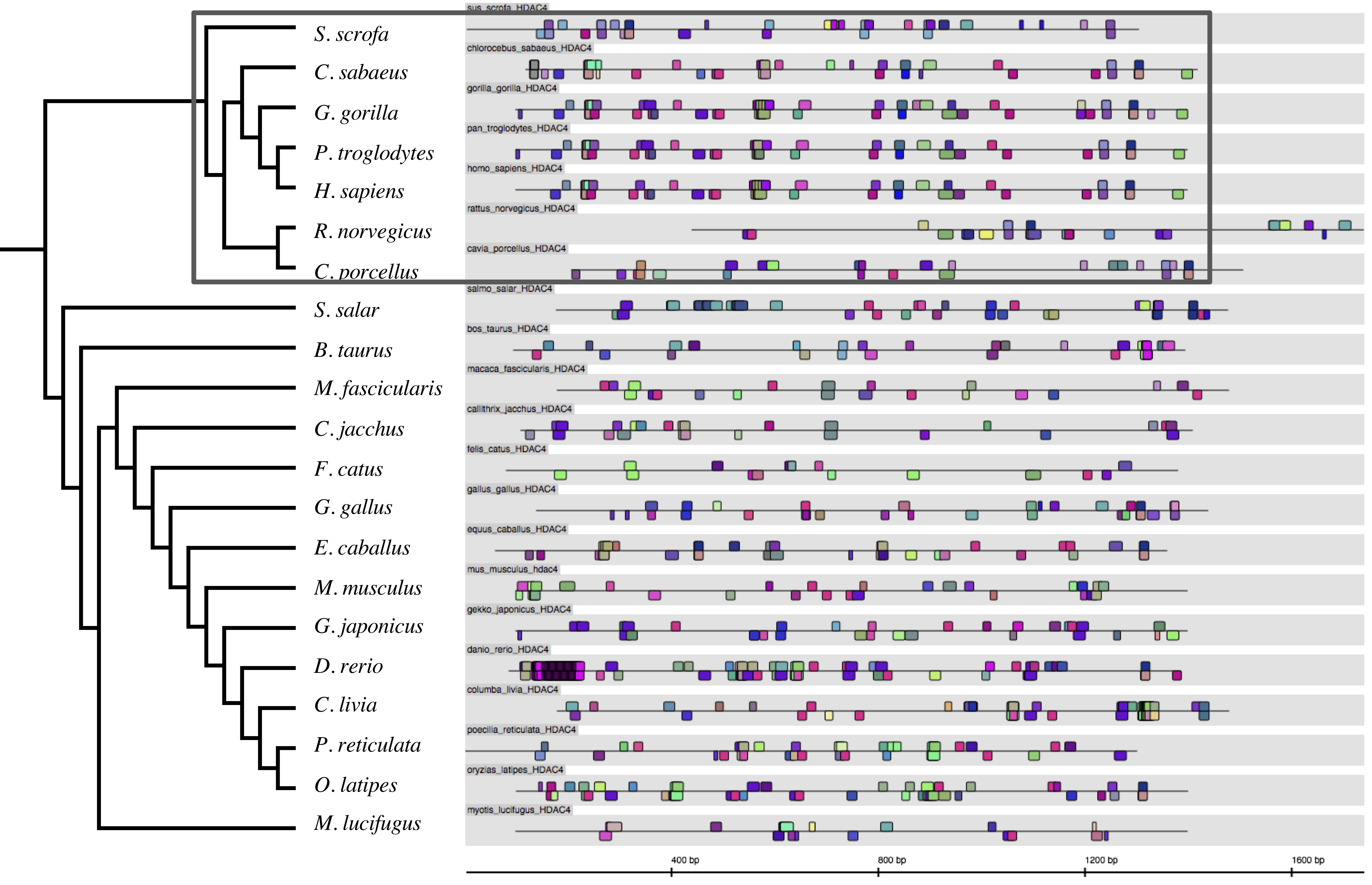
**

**Figure S3.** Evolutionary conservation of transcription factor binding sites in HDAC4 promoter sequences in different organisms.
