# Supplementary material for: Promoter conservation in HDACs points to functional implications
Source: BMC Genomics. 2019 Jul 27;20:613. doi: 10.1186/s12864-019-5973-x (PMC6660948; doi:10.1186/s12864-019-5973-x)
Supplement: Supplementary file 7 — : Figure S6 Evolutionary conservation of transcription factor binding sites in HDAC9 promoter sequences in different organisms. (DOCX 3052 kb) [file 12864_2019_5973_MOESM7_ESM.docx]

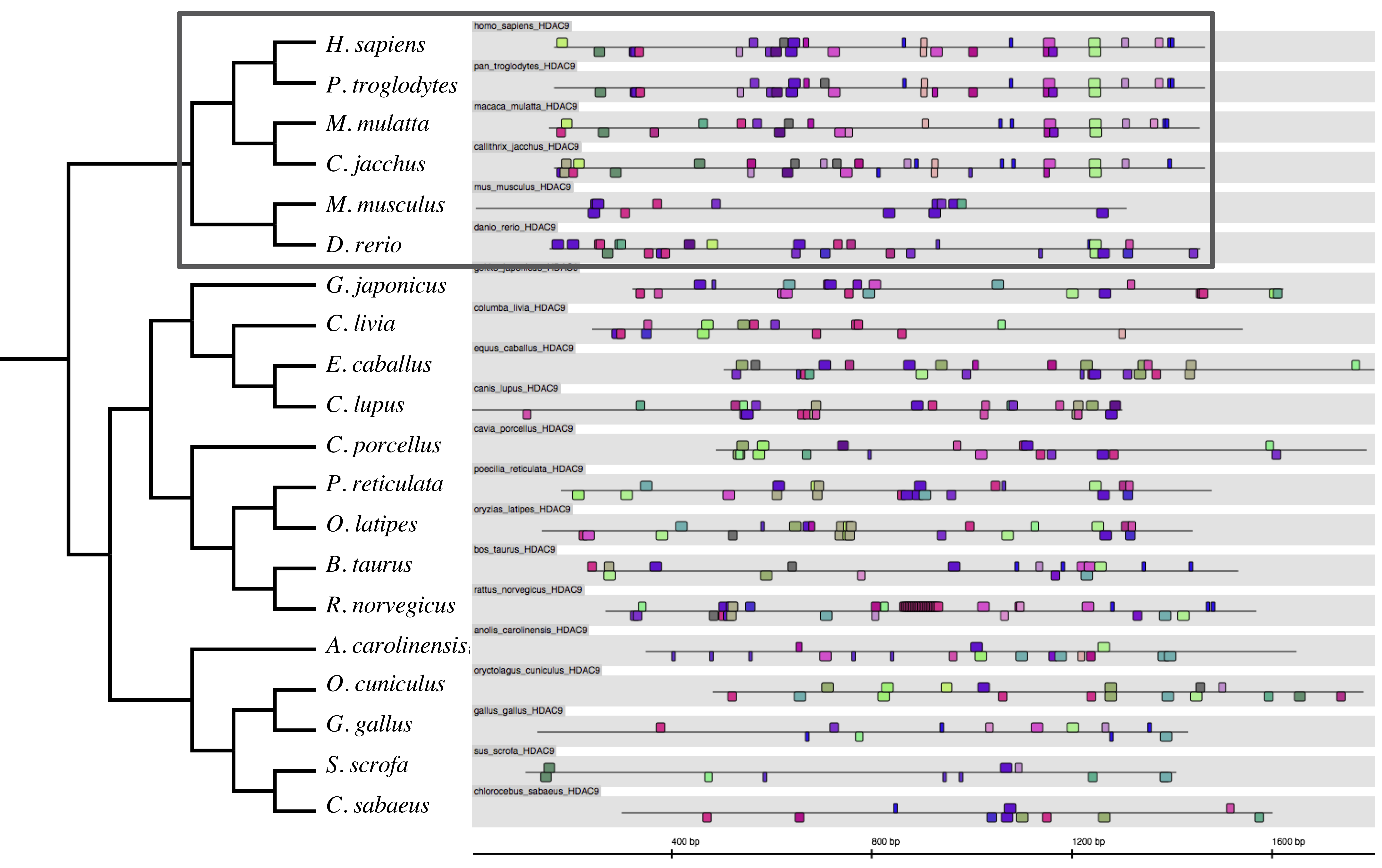


**Figure S6.** Evolutionary conservation of transcription factor binding sites in HDAC9 promoter sequences in different organisms.
