# Supplementary material for: Promoter conservation in HDACs points to functional implications
Source: BMC Genomics. 2019 Jul 27;20:613. doi: 10.1186/s12864-019-5973-x (PMC6660948; doi:10.1186/s12864-019-5973-x)
Supplement: Supplementary file 8 — : Figure S7 Evolutionary conservation of transcription factor binding sites in HDAC11 promoter sequences in different organisms. (DOCX 3544 kb) [file 12864_2019_5973_MOESM8_ESM.docx]

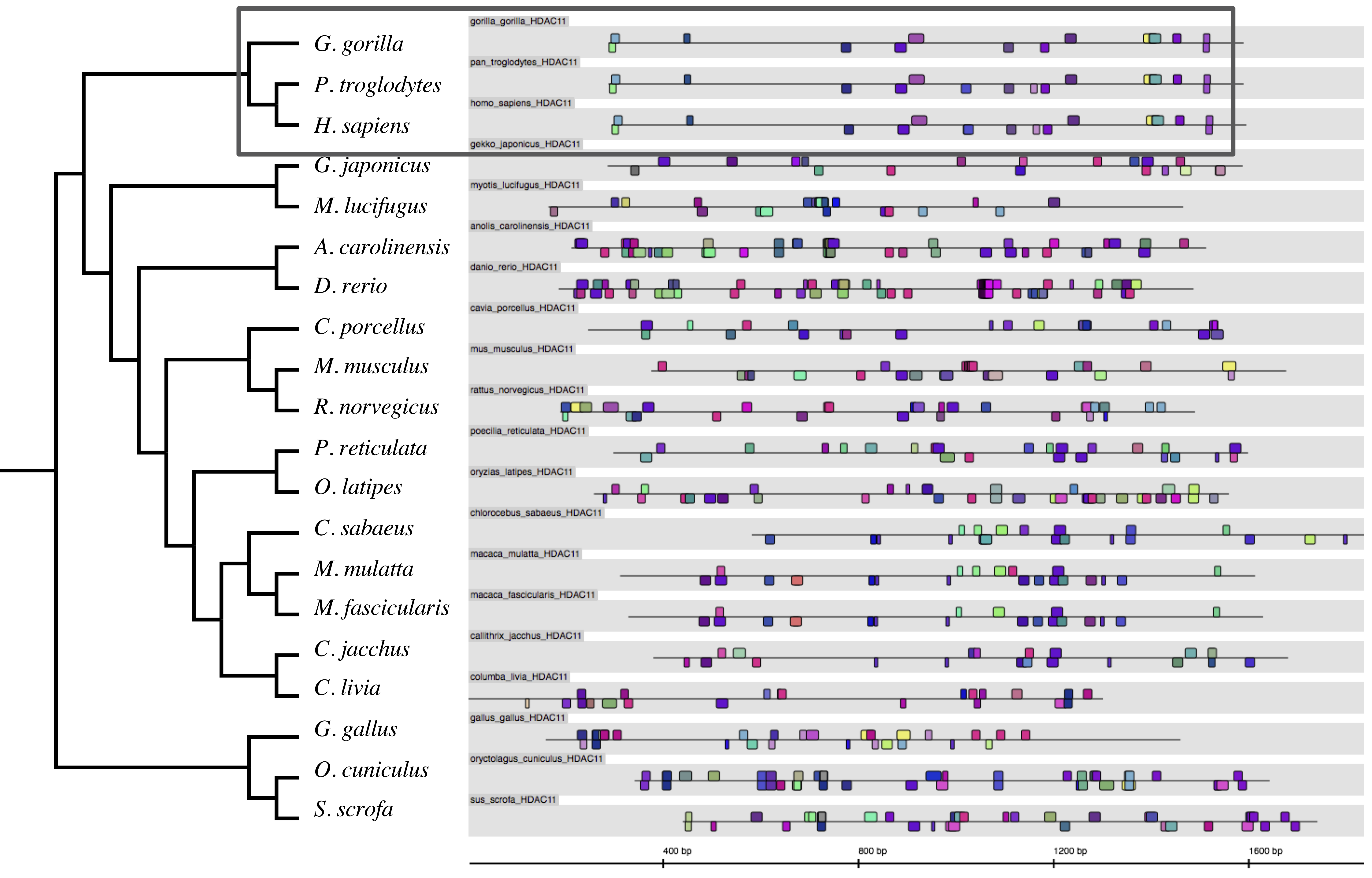


**Figure S7.** Evolutionary conservation of transcription factor binding sites in HDAC11 promoter sequences in different organisms.
